# Fabricating a SFMA/BAChol/PAA/ZnCl_2_ Hydrogel with Excellent Versatile Comprehensive Properties and Stable Sensitive Freezing-Tolerant Conductivity for Wearable Sensors

**DOI:** 10.3390/ijms252413339

**Published:** 2024-12-12

**Authors:** Jie-Ping Fan, Ming-Ru Xie, Chao Yuan, Jia Ma, Ke-Pu Fu, Chun-Hong Huang, Hui-Ping Chen, Hai-Long Peng, Chun-Fang Xie

**Affiliations:** 1 Department of Chemical Engineering, School of Chemistry and Chemical Engineering, Nanchang University, Nanchang 330031, Chinac.xie@ncu.edu.cn (C.-F.X.); 2 Key Laboratory of Poyang Lake Ecology and Bio-Resource Utilization of Ministry of Education, Nanchang University, Nanchang 330031, China; 3 School of Basic Medical Sciences, Nanchang University, Nanchang 330006, China

**Keywords:** flexible wearable sensor, high mechanical strength, versatile comprehensive functionalities, sensitive electrical conductivity, molecular mechanism

## Abstract

Flexible wearable sensors have obtained tremendous interest in various fields and conductive hydrogels are a promising candidate. Nevertheless, the insufficient mechanical properties, the low electrical conductivity and sensitivity, and the limited functional properties prevent the development of hydrogels as wearable sensors. In this study, an SFMA/BAChol/PAA/ZnCl_2_ hydrogel was fabricated with high mechanical strength and versatile comprehensive properties. Specifically, the obtained hydrogel displayed excellent adhesion and mechanical stability, cryophylactic ability, stable sensitive freezing-tolerant conductivity, and feasible electrical conduction under a wide temperature range, demonstrating the high application potential as a flexible wearable sensor for movement behavior surveillance, even under harsh environments. Furthermore, the mechanical strength of the hydrogel could easily be regulated by varying the copolymer content. The molecular mechanisms of the hydrogel formation and the reversible adhesion during the wet-dry transition were proposed. The non-covalent interactions, including the electrostatic interaction, hydrogen bond interaction and hydrophobic association, and coordination interaction, were dynamically presented in the hydrogel network and hence supported the versatile comprehensive properties of the hydrogel. This study provides a strategy for designing novel hydrogels to promote the development of flexible sensors with stable sensitive freezing-tolerant conductivity.

## 1. Introduction

With the fast development of artificial intelligence technology, wearable flexible sensors have obtained significant attention in recent years. In particular, due to the high water content and tissue-similar physical properties, hydrogels and hydrogel-based composites have been widely used as smart wearable electronics in the past several decades [1,2]. Hydrogels are a kind of three-dimensional and cross-linked polymeric network with hydrophilic, softer, porous, tunable mechanical properties, consistent stimulus responsiveness, etc. [3,4,5]. The intrinsic flexibility of the hydrogels is highly suited for integration with soft biological tissues, contributing to maintaining stable signals under rigorous movements and deformation during human motion [6]. However, the limited mechanical properties, low electrical conductivity and sensitivity, and poor wearing comfort have prevented the development of hydrogels as wearable sensors. Converting biological signals efficiently and accurately to electrical signals in different states and environments is of great significance for wearable sensors. Practically, the balance of mechanical properties (high strength and high toughness) and electrical conductivity (high conductivity and sensor sensitivity) is quite difficult [7]. The addition of metal nanoparticles [8], carbon nanotubes [9], and other conductive materials into the hydrogel network can improve the hydrogel conductive sensitivity, but it always results in the heterogeneity of the hydrogel networks and hence reduces the hydrogel’s mechanical strength and conductive stability. Furthermore, their use as wearable sensors requires the hydrogel with diverse functionalities of sufficient adhesiveness, self-healing abilities, biocompatibility, antibacterial activity, and so on [10,11]. Unfortunately, the present hydrogels always display limited functional properties [12]; it is still challenging to obtain hydrogels with excellent versatile comprehensive properties for wearable sensor applications.

Over the past years, many strategies have been employed for improving hydrogels’ mechanical and functional properties. The introduction of reversible interactions in the hydrogel network has been found to greatly increase the mechanical properties [13], such as hydrogen bonds, metal coordination bonds, dynamic covalent interactions, and hydrophobic interactions [10,14]. Specifically, the dynamic covalent interactions and the hydrophobic interactions in the hydrogel network greatly contribute to energy dissipation, self-healing, and fatigue resistance [15]. In addition, the application of metal coordination in fabricating smart hydrogels has made considerable progress; the incorporation of Fe^3+^, Ca^2+^, Zn^2+^, and Ag^+^ into the hydrogel to form new physical crosslinking points can vastly increase the conductivity and sensitivity, as well as the mechanical strength [5,16]. A combination of metal coordination, covalent interactions, and hydrophobic interactions has great potential for both improving mechanical properties and facilitating diverse functions [17].

Silk fibroin (SF), which is a natural polymer protein extracted from silk with excellent biocompatibility, controllable biodegradability, and adjustable mechanical properties, is widely used in the biological materials of hydrogels, films, and three-dimensional scaffolds, etc. [18,19]. In our previous studies, the SF or vinyl-modified SF (SFMA) was used to prepare hydrogels with high biocompatibility toward proteins, and the SF/SFMA was also found to be able to adjust the mechanical strength of the obtained hydrogel [20,21]. Besides SF/SFMA, acrylic acid (AA) is also a commonly used monomer to produce hydrogels with a high water-absorbing capacity, which is always combined with some other polymers to produce different forms of hydrogels [22]. Poly(acrylic acid) (PAA) hydrogel, a biocompatible and biodegradable polymer, has attracted remarkable interest in the fields of tissue engineering, protein immobilization, biosensors, etc. [23]. For both AA and SF, the abundance of carboxyl groups and amino groups in their molecules contributes to the production of many covalent interactions and hydrophobic interactions [24,25].

In this study, using the SFMA and PAA as the skeletons, an SFMA/BAChol/PAA/ZnCl_2_ hydrogel was fabricated with good mechanical properties. A variety of hydrogel functional properties were also investigated; the molecular mechanisms of the hydrogel formation and the reversible adhesion during the wet–dry transition were proposed. Owing to the multi-reversible interactions among AA, SFMA, BAChol, and Zn^2+^; the ion migration channels from Zn^2+^; and the antibacterial performance of the BAChol and ZnCl_2_, the fabricated SFMA/BAChol/PAA/ZnCl_2_ hydrogel displayed multifunctional performance with high toughness, strong adhesiveness, self-healing performance, and excellent fatigue resistance, as well as good biocompatibility, antibacterial activity, good swelling, and moisture retention ability. Specifically, the obtained hydrogel presented stable freezing-tolerant conductivity, and feasible electrical conduction under a wide temperature range, demonstrating high application potential as wearable sensors.

## 2. Results

### 2.1. Effect of the Preparation Conditions on the Hydrogel Mechanical Strength

The mechanical properties of hydrogels are of vital importance for their application as wearable sensors. The poor mechanical strength, which always results from heterogeneity and/or high water content, seriously limits their application [26,27]. In this study, the mechanical strength under different preparation conditions was investigated; the amounts of SFMA, AA, BAChol, glycerol, and ZnCl_2_ were optimized; both the strain and the stress were increased and then decreased by increasing their amounts; the strain was varied from 254% to 1482%; and the stress was varied from 0.075 MPa to 0.19 MPa. The optimal preparation conditions were 50 mg/mL of SFMA, 25 mg of BAChol, 0.6 mL of AA, 0.360 mL of glycerol, and 8 mg/mL ZnCl_2_, with 8 mg of AAPH in every 2.5 mL of batch solution mixture. Under these conditions, the mechanical strength of the obtained hydrogel was achieved at around 1.61 MPa at 80% compression. Prior to the compression break, the hydrogel showed good resilience. During the tensile test, the maximum tensile strength of the obtained hydrogel was achieved at around 0.145 MPa, and the ultimate elongation at the breaking point was around 1580%. The results demonstrated that the obtained hydrogel had excellent elasticity, high compressive strength, and superstretchability, which was well-suited to wearable sensor applications. A detailed description of all results and the explanation, together with the photos and the stress–strain curves, which depicted a straightforward observation of the compression and tensile tests, can be found in the Supporting Information (Section S1).

### 2.2. Toughness and Fatigue Resistance of the Obtained Hydrogel

**Figure 1:**
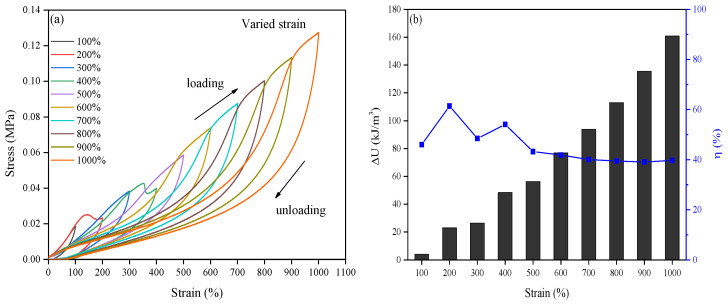

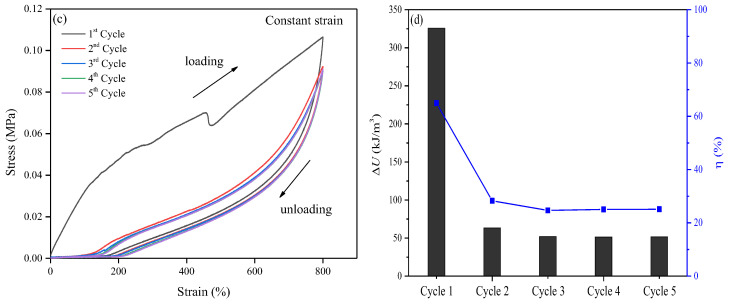
Consecutive loading–unloading tensile test of the same hydrogel. (**a**) Stress–strain curve of the hydrogel in the cyclic loading–unloading tensile tests under different maximum loading strains from 100% to 1000%; (**b**) hysteresis energy (dissipated energy) (Δ*U*) and energy loss coefficient (*η*) in each loading–unloading cycle under different maximum loading strains from 100% to 1000%; (**c**) stress–strain curve of the hydrogel in the cyclic loading–unloading tensile tests under a 800% maximum loading strain; (**d**) hysteresis energy (dissipated energy) (Δ*U*) and energy loss coefficient (*η*) in each loading–unloading cycle under a 800% maximum loading strain.

Wearable sensors are constantly subjected to multiple cycles of stretching or bending during their applications, which requires the sensors to have good toughness and fatigue resistance to dissipate energy, preclude structural damage, etc. [28,29,30,31]. In this study, to investigate the toughness of the obtained hydrogel, the cyclic loading–unloading tests with different maximum loading strains varying from 100% to 1000% were consecutively performed on the same hydrogel without intervals. The results are shown in Figure 1a; the presence of the hysteresis loops was related to the partial rupture of the non-covalent interactions in the hydrogel network, which was common in numerous similar systems with non-covalent interactions [32]. A larger hysteresis loop was obtained at a higher maximum strain, indicating a higher energy dissipation degree by increasing the strain, which was because more non-covalent interactions were ruptured at a higher loading strain [32,33]. Furthermore, the superposition of the successive loading–unloading cycles was observed, indicating that the dissipated energy was partially irreversible, which resulted from the covalent bond rupture and was analogous to the Mullins effect [34,35]. Even so, the hydrogel was able to recover quickly in each stretching, thanks to the quick recovery of the broken hydrogen bonds, hydrophobic associations, and Zn^2+^ coordination [31]. The results demonstrated the good fatigue resistance of the hydrogel.

On the basis of the hysteresis curves, the hysteresis energy (dissipated energy) in each loading–unloading cycle (Δ*U*, kJ/m^3^) and the energy loss coefficient (*η*, %) were calculated according to the calculation methods described in the literature [32,36,37]. The results are shown in Figure 1b; the dissipated energy increased quickly by increasing the strain. The dissipated energy was about 4.0 kJ/m^3^ at 100% strain, indicating that the hydrogel mainly underwent elastic deformation; the rupture of the hydrogen bonds, the hydrophobic associations, and the Zn^2+^ coordination was negligible at the 100% strain [38]. However, the dissipated energy was significantly improved to 160 kJ/m^3^ at the 1000% strain, providing efficient energy dissipation to toughen the obtained hydrogel [34]. By contrast, the energy loss coefficient was around 40% for every cycle except the initial ones, demonstrating the obtained hydrogel dissipating the energy with around the same efficiency and showing good fatigue resistance [32,35]. This was because the non-covalent interactions in the hydrogel network were considerably broken at the initial stretching, and the network was weakened and could be immediately recovered during the following cycles [32,35].

Furthermore, the cyclic loading–unloading tests were consecutively performed on the same hydrogel without intervals at the maximum loading strain of 800%. As indicated in Figure 1c, a large hysteresis loop was observed in the first cycle, indicating the good energy dissipation ability of the hydrogel; this was attributed to the disassociation of multiple hydrogen bonds, covalent bonds, hydrophobic associations, and Zn^2+^ coordination within the hydrogel network. In the following cycles, the hysteresis loops were much smaller than the first one, which was analogous to the Mullins effect [34,35]. The hysteresis loop in the first cycle was larger than those in the rest cycles, which was attributed to the irreversible rupture of chemical crosslinks formed between SFMA and PAA chains through covalent bonds and the self-crosslinks in SFMA chains and PAA chains. By contrast, the broken hydrogen bonds, hydrophobic associations, and Zn^2+^ coordination were able to recover in each stretching, contributing to almost the same hysteresis loop observed in the following successive stretching cycles. On the basis of the dissipated energy and the energy loss coefficient results, as indicated in Figure 1d, a significant energy dissipation of around 325 kJ/m^3^ with an energy loss coefficient of about 65% was observed in the first tensile cycle. In the succeeding cycles, the dissipated energy and the energy loss coefficient were stable at around 50 kJ/m^3^ and 25%, respectively. This further demonstrated that the hydrogel network was weakened with non-covalent bond breakage at the beginning, and the broken non-covalent interactions could not be immediately recovered during the following cycles, leading to the lower, stable dissipated energy and energy loss coefficient [32]. The results demonstrated the superstretchability, high toughness, excellent elasticity, good stretchability, and excellent fatigue resistance performance of the hydrogel.

### 2.3. Adhesive Performance of the Obtained Hydrogel

**Figure 2:**
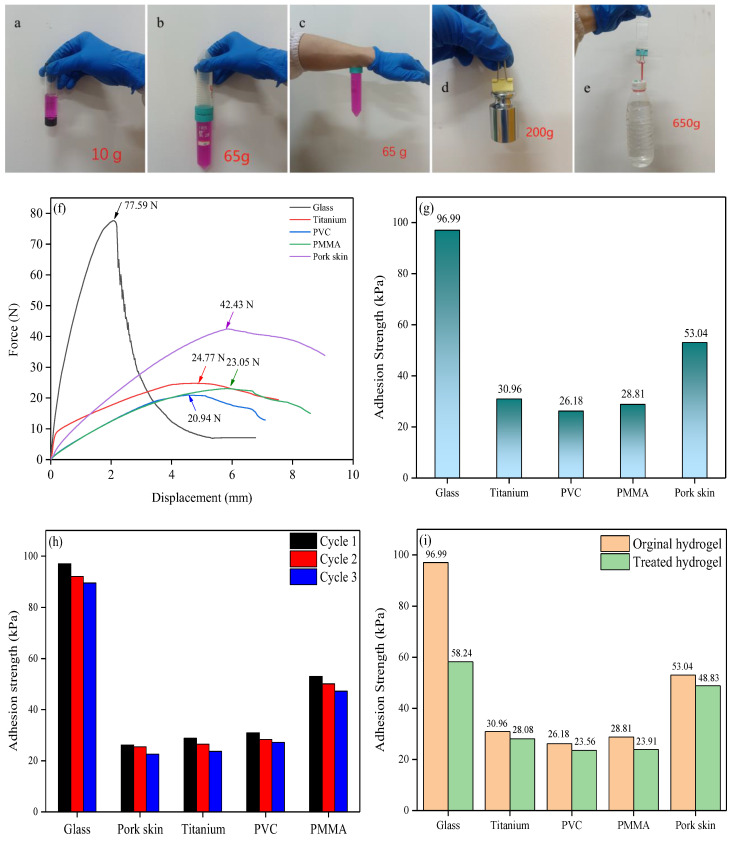
Adhesive performance of the obtained hydrogel applied to various substrates: (**a**) glass bottle, (**b**) PTFE centrifuge tube, (**c**) skin and PTFE, (**d**) stainless steel weight, and (**e**) glass sheet; (**f**) and (**g**) quantitative characterization of the adhesion strength for the hydrogel applied to various substrates; (**h**) reusable adhesion performance of the obtained hydrogel on the adhesion strength toward different substrates during the cyclic contact–separate process; (**i**) reversible adhesion performance during the repeated lap shear test of the obtained hydrogel to different substrates.

The adhesive behavior of the obtained hydrogel was visualized using the hydrogel sample to glue the substrates fully together with the help of 5 N pressure for 30 min. The hydrogel slice was used at an interface of 40 mm length and 20 mm width. The substrates used were glass, a PTFE centrifuge tube, skin, and a stainless steel weight. Prior to the test, the substrate surface was washed with ethanol and then deionized water and wiped dry. The results are shown in Figure 2a–e; the hydrogel could firmly glue a wide range of materials together and displayed good adhesive properties. The adhesive performance was further quantitatively investigated using the tensile tests at a 10 mm/min speed, following typical lap shear tests (GB/T 7124-2008 [39]) on different substrates. The adhesion strength was calculated by the measured maximum force divided by the interface area [33]. The results are shown in Figure 2f,g. The obtained hydrogel displayed high performance on all the tested substrates; the highest adhesion strength was achieved on glass, which was as high as 96.99 kPa. Furthermore, the adhesion strength of the obtained hydrogel toward pork skin (mimicking human skin) was also up to 53.04 kPa, indicating the huge application potential of the obtained hydrogel as wearable sensors.

A cyclic contact–separate process was performed to investigate the reusable performance of the obtained hydrogel on the adhesion strength toward different substrates. The results are shown in Figure 2h; the adhesion strength was almost the same during the cyclic tests for all the investigated substrates. The reversible adhesion performance of the obtained hydrogel to different substrates was also investigated. The obtained hydrogel was freeze-dried and immersed in water for 1 h (treated hydrogel), and then it was subjected to the lap shear test. The results are shown in Figure 2i. Compared with the initial adhesion performance, the treated hydrogel displayed only a slight decrease in adhesion strength for the titanium sheet, PVC, PMMA, and pork skin, even though the adhesion strength of the treated hydrogel toward glass significantly decreased. In particular, the treated hydrogel still achieved an adhesion strength of 48.83 kPa toward pork skin. Furthermore, during the investigation, the dried hydrogel was found to decrease drastically in adhesion performance, and it could not adhere to any of the investigated materials. The results demonstrated the good dynamic and reversible adhesion performance of the obtained hydrogel, that is, the high adhesion strength of the fresh hydrogel, and the low adhesion strength of the dried hydrogel.

### 2.4. Self-Healing Performance of the Obtained Hydrogel

**Figure 3:**
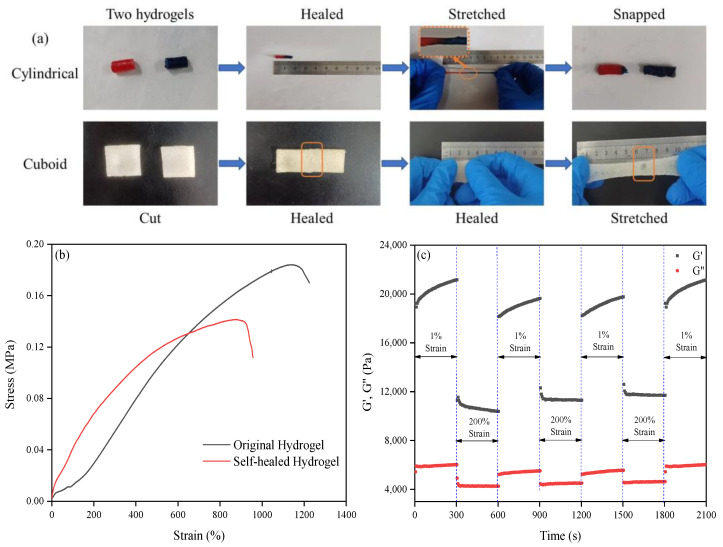

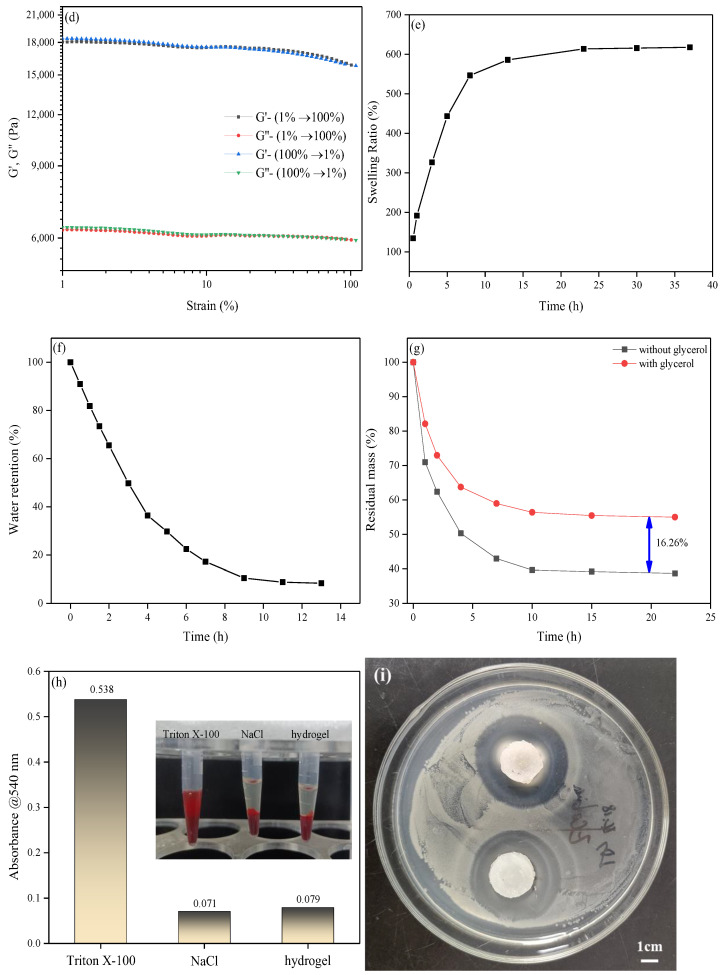
(**a**) Self-healing process of the obtained hydrogel; (**b**) comparison of the stress–strain curve of the original obtained hydrogel and the self-healed hydrogel; (**c**) storage modulus (G′) and loss modulus (G″) of the hydrogel under strain alternation tests between 1% and 200% at 25 °C and 1 Hz frequency; (**d**) storage modulus (G′) and loss modulus (G″) of the hydrogel under strain varying from 1% to 100% and then back to 1% at a frequency of 1 rad/s and 25 °C; (**e**) swelling ratio of the obtained hydrogel under different time; (**f**) water retention ability of the obtained hydrogel under different times; (**g**) comparison of the moisture retention ability between the hydrogel with and without glycerol; (**h**) supernatant absorbance of the different incubation solutions indicating the hemolytic activity of the obtained hydrogel; (**i**) inhibition zone of the *E. coli* cells’ proliferation performance in the presence of the hydrogel obtained in this study.

The self-healing performance of the obtained hydrogel was investigated by both macroscopic observation and tensile tests. For macroscopic observation, two hydrogels were put in a plastic bag for 6 h at room temperature without any irritation. Either, a cuboid hydrogel was cut from the middle into two halves, and the resulting two pieces were placed with the cut surfaces together in a plastic bag for 6 h at room temperature without any irritation. Then the healing hydrogel was manually stretched to the snapped state. For convenience, the two cylindrical hydrogels were dyed in different colors during preparation. The results are shown in Figure 3a. The self-healed hydrogels were not snapped when they were stretched to four fold the original length. By further stretching, when they were pulled off, the fracture surface was obviously not the adhesion interface. By further investigating the mechanical strength, Figure 3b indicates that the self-healed hydrogel regained 76.6% and 78.2% of the original stress and strain, respectively.

The self-healing property of the obtained hydrogel was further investigated by applying the original hydrogel (30 mm in diameter and 1 mm in thickness) to the strain alternation tests with the help of a TA Discovery Hybrid Rheometer. The results are shown in Figure 3c. At the beginning, the hydrogel was under 1% strain for 300 s at 25 °C and 1 Hz frequency; both the storage modulus (G′) and loss modulus (G″) were at a relatively high value. When the hydrogel was under 200% strain, G′ and G″ decreased sharply, indicating the breakage of the non-covalent interactions. However, when the strain returned to 1%, both moduli immediately recovered to the initial level. This strain alternation was repeated several times, and G′ and G″ maintained a stable trend. Furthermore, at the frequency of 1 rad/s and 25 °C, the obtained hydrogel was stretched by varying the strain from 1% to 100% and then back to 1%, and the modulus curves were almost superimposed during the cyclic strain changing, as indicated in Figure 3d, indicating the fast recoverability of the hydrogel network.

The results demonstrated the excellent self-healing properties of the obtained hydrogel; the non-covalent interactions in the hydrogel network were broken when stretching but were rapidly recovered when the strain was decreased. This reversible dissociation and reformation of the dynamic cross-linked networks greatly contributed to the super-fast self-healing performance of the obtained hydrogel [30,40].

### 2.5. Swelling and De-Swelling Ability, Moisture Retention Ability, Hemolytic Activity, and Antibacterial Activity

To investigate the swelling ability of the obtained hydrogel, the freeze-dried hydrogel was immersed in PBS at 37 °C until it reached the swollen state, and the swelling ratio at different time intervals was calculated by determining the degree of mass change before and after swelling. For the shrinking ability, on the contrary, the hydrogel in the swollen state was put into a 37 °C drying oven, and the shrinking ratio was calculated by dividing the mass at different drying time intervals by the initial mass. The results are shown in Figure 3e,f. During swelling, the swelling speed was rapid at the initial 8 h and then slowed down from the 8th hour until it almost reached the swollen state at around 23 h; the equilibrium swelling ratio was around 618%. For de-swelling, a de-swelling ratio as high as 62% was achieved at the initial 4 h; from then, the de-swelling speed decreased until reaching equilibrium at around 11 h.

To investigate the moisture retention ability, the fresh hydrogel was put into a 37 °C drying oven for different time intervals, and the moisture retention ability was calculated by dividing the mass at different drying time intervals by the initial mass. Note that the shrinking ratio was investigated using the swollen hydrogel, but the moisture retention ability was investigated using the fresh hydrogel. For comparison, the hydrogel prepared without glycerol was used as the blank control. The results are shown in Figure 3g. The hydrogel without glycerol decreased much faster in weight than the hydrogel with glycerol. Within the first hour, the weight loss of the hydrogel without glycerol was as high as 29.02%, while the hydrogel with glycerol was only 18.21%. Furthermore, both hydrogels achieved equilibrium 10 h later, at which point the weight loss was at around 61.27% for the hydrogel without glycerol but only 45.01% for the hydrogel with glycerol. The results demonstrated the high moisture retention ability of the obtained hydrogel in this study; adding glycerol contributed to increasing the hydrogel moisture retention ability, which was in favor of slowing down the hydrogel’s aging during use.

The hemolytic activity of the obtained hydrogel was investigated by adding 100 mg hydrogel into 1 mL of an erythrocyte solution (in 0.9% NaCl) and incubating the solution at 37 °C for 3 h. Subsequently, the erythrocyte solution was centrifuged at 2500 rpm for 5 min, the supernatant was detected using UV–Vis spectroscopy (TU-1901, Persee, Beijing, China) at 540 nm. For comparison, the negative control was added 100 mg of 0.9% NaCl but without adding hydrogel. The positive control was added 100 mg 0.1% Triton X-100 instead of the hydrogel. The results are shown in Figure 3h. It is clear that the obtained hydrogel did not result in erythrocyte hemolysis, showing good biocompatibility, which is important for the hydrogel to be used for wearable electronics.

To investigate the antibacterial activity of the obtained hydrogel, the *E. coli* suspension (around 1 × 10^8^ CFU/mL) was inoculated on the solid agar medium with a hydrogel slice and then incubated for 10 h at 37 °C. The proliferation performance of the *E. coli* cells was recorded, and the bacterial inhibition zone was calculated with the help of ImageJ software (Version 1.53e). Prior to investigation, the hydrogel slice was sterilized under UV light for 30 min. The results are shown in Figure 3i; an inhibition zone was clearly visualized, and the area was about 19.63 cm^2^. The results demonstrated the good antibacterial activity of the obtained hydrogel. The presence of BAChol improved the metabolic activity to produce more reactive oxygen species that damaged the bacterial integrity, and it also was able to prevent peptidoglycan formation to inhibit bacterial activity [41]. The Zn^2+^ in the hydrogel network also generated reactive oxygen species to destroy bacterial fertility [42,43].

### 2.6. Electrical Conduction Performance of the Obtained Hydrogel

**Figure 4:**
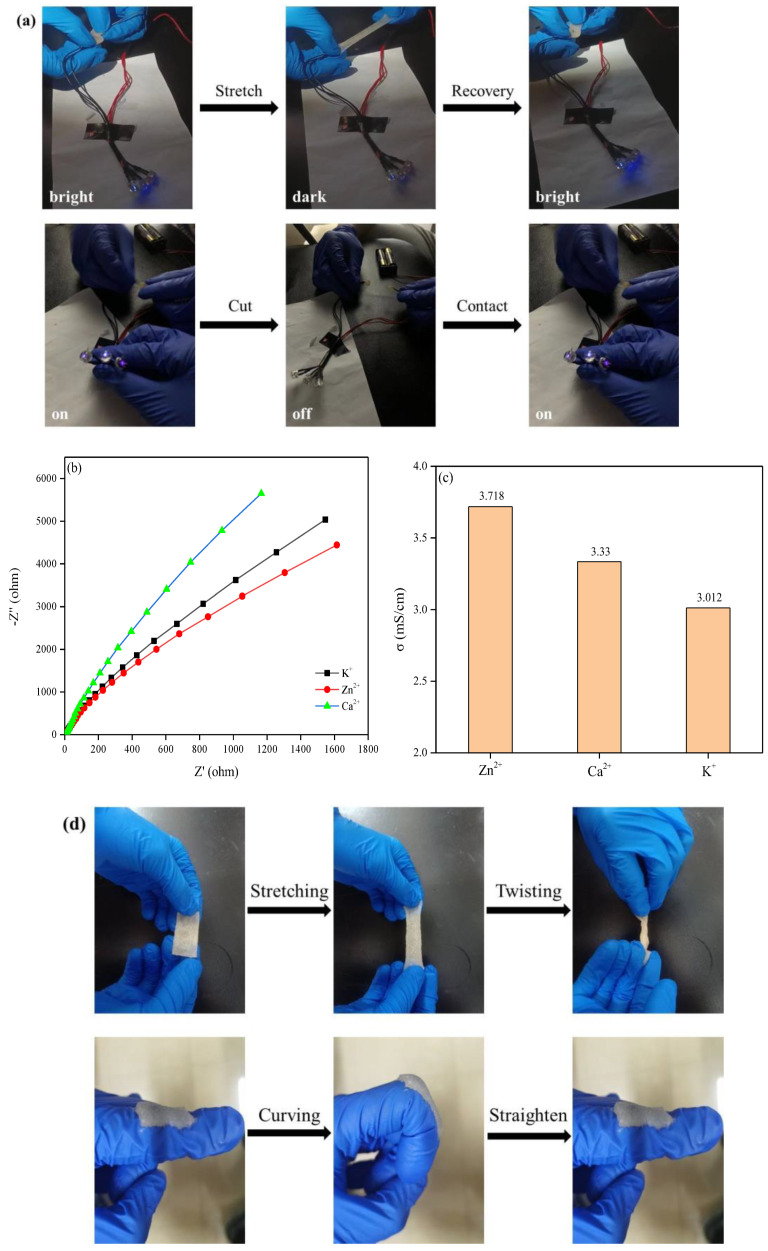

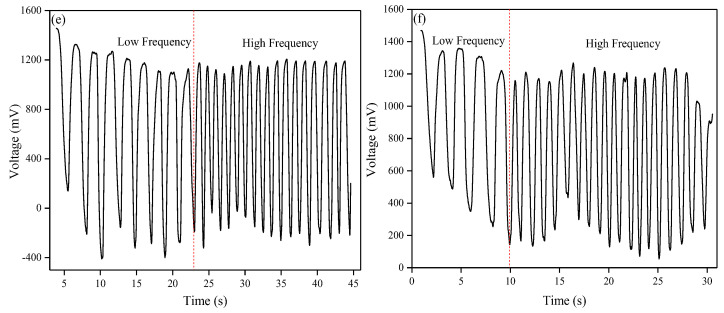
Electrical conduction behavior of the obtained hydrogel; (**a**) implementation in an LED bulb; (**b**) Nyquist curve comparison after substituting Zn^2+^ with Ca^2+^ or K^+^; (**c**) conductivity comparison after substituting Zn^2+^ with Ca^2+^ or K^+^; (**d**) mechanical and adhesion performance following pre-treatment at −80 °C; (**e**) voltage signal of the hydrogel pre-treated at 25 °C under different frequencies of finger movement; (**f**) voltage signal of the hydrogel following pre-treatment at −80 °C under different frequencies of finger movement.

The electrical conduction behavior of the obtained hydrogel was visualized using an LED bulb, the results are shown in Figure 4a. The illumination of the LED bulb indicated the conductive nature of the obtained hydrogel. When stretching the hydrogel, the bulb became darker, but it became bright again when the hydrogel was recovered to its initial length, indicating the change of electrical resistance during stretching. Furthermore, the LED bulb went out when the hydrogel was cut, but it was bright again when the two halves of the hydrogel were in contact. The results indicated that the obtained hydrogel displayed good electrical conduction performance as well as good self-healing performance.

Furthermore, the electrical conduction performance was investigated using an electrochemical workstation (CHI660E, Shanghai, China). Following recording the real-time *U*-*I* curve, the electrical resistance was calculated using Ohm’s law, and then the conductivity was calculated according to the length and sectional area of the tested hydrogel. The results indicated that the conductivity of the obtained hydrogel in this study was as high as 3.718 mS/cm. The Zn^2+^ coordination interactions, which were formed with the –COOH groups from PAA, the –OH groups from BAChol, and the –NH_2_ groups and –OH groups from SFMA, enabled the electrical conductivity of the obtained hydrogel [31]. When further substituting the Zn^2+^ by Ca^2+^ or K^+^, for comparison, the Nyquist curve was obtained, as shown in Figure 4b,c; the results showed the highest electrical conduction performance of the obtained hydrogel in this study, which highlighted the superiority of the hydrogel obtained in this study.

Subsequently, the obtained hydrogel was used to monitor finger joint movement; the hydrogel adhered to the finger, and the voltage was detected by the electrochemical workstation. It is noteworthy that many softer materials would become stiff and fragile when frozen under low temperatures, resulting in a drastic reduction in adhesion and conductivity [44]. In this case, the investigation was performed following the treatment of the hydrogel for 24 h at both 25 °C and −80 °C. The mechanical and adhesion performance of the hydrogel treated at −80 °C are shown in Figure 4d. The electrical conduction sensitivity of the hydrogel at both 25 °C and −80 °C under different frequencies of finger movement is shown in Figure 4e,f. During stretching and twisting, the hydrogel still showed good mechanical strength. When curving, the hydrogel was tightly bound to the finger all the time. The voltage signal was almost the same and showed no obvious attenuation for the hydrogel treated at both 25 °C and −80 °C. Furthermore, the voltage signal was sparse at a low curving frequency while a dense voltage signal was detected at a high curving frequency. The results demonstrated the cryophylactic ability and mechanical stability of the hydrogel. The hydrogel displayed a stable sensitive freezing-tolerant conductivity and a feasible electrical conduction under a wide temperature range. The freezing-tolerant conductivity contributes to the use of the obtained hydrogel for high-performance supercapacitors and batteries, soft actuators, cryogenic sensors, etc. [45,46]. The results confirmed the obtained hydrogel in this study to be a promising wearable sensor for movement behavior surveillance. The presence of glycerol, which can generate abundant hydrogen bonds with H_2_O molecules and lock these H_2_O molecules within the hydrogel network, contributed to moisture retention [47,48]. The coordination interaction from Zn^2+^ and the hydrogen bonds of the surrounding hydration layer weakened the aging and stiffening of the hydrogel. In this case, the hydrogel improved in its mechanical strength and adhesion ability, as well as its electrical conductivity and sensitivity. Critically, the investigation related to −80 °C only pre-treated the hydrogel at −80 °C for 24 h and then immediately tested it at room temperature.

## 3. Discussion

Polymer hydrogels are a kind of three-dimensional polymer network hydrogel widely used as flexible wearable sensors [49,50]. The application of the hydrogel is still greatly limited because of insufficient mechanical strength, low conductivity sensitivity, and limited functional properties [29]. This study synthesized an SFMA/BAChol/PAA/ZnCl_2_ polymer hydrogel with good mechanical properties and a stable sensitive conductivity to be used for flexible wearable sensors. Furthermore, the mechanical strength of the hydrogel could easily be regulated by varying the copolymer content (Section S1 in the Supporting Information), which largely increased the flexibility of the obtained hydrogel for wearable sensors. Furthermore, the obtained hydrogel displayed versatile comprehensive properties, contributing to its use in flexible sensors [51], such as biocompatibility, moisture retention ability, strong and reversible adhesion, reliable self-healing capability, and antibacterial activity. Importantly, the obtained hydrogel maintained its mechanical strength, adhesion ability, and stable sensitive conductivity under a wide temperature range, greatly broadening its wearable sensor application under harsh environments. Meanwhile, the biocompatibility and antibacterial activity of the obtained hydrogel also enable it to be promising for wound dressing, which greatly expands the application fields and highlights the value of the hydrogel obtained in this study.

In recent years, many works have been devoted to preparing a polymer hydrogel with good mechanical strength and strong adhesion. Yan et al. [52] synthesized a glycerol-ionic hybrid hydrogel using acrylamide monomer and branched polyethylenimine, which achieved 5.4 kPa stress, 15,000% strain, and 6 kPa adhesion strength at 25 °C. Fan et al. [53] prepared a poly(1-vinylimidazole)-choline chloride-glycerol eutectogel using a deep eutectic solvent formed with choline chloride and glycerol, which achieved 490 kPa stress, 2310% strain, and 13.6 kPa adhesion strength. Yin et al. [54] fabricated a self-adhesive conductive chitosan/tannic acid/poly(acrylic acid)-Al^3+^ ionic hydrogel sensor by a facile one-pot free radical polymerization, with 179.3 kPa stress, 1450% strain, 7.02 kPa adhesion strength. Mo et al. [55] also prepared a tannic acid-enabled dynamic interactions hydrogel by using acrylic acid, (3-acrylamidophenyl) boronic acid, and CaCl_2_; the hydrogel displayed 54 kPa stress, 7300% strain, 50 kPa adhesion strength. Shui et al. [56] synthesized an oligo-polydopamine-tyrosine-poly(vinyl alcohol) hydrogel by incorporating tyrosine–dopamine conjugates as dynamic cross-linkers into the poly(vinyl alcohol) matrix, achieving 13,300 kPa stress, 813% strain, 42.1 kPa adhesion strength. Wang et al. [57] synthesized a zwitterionic polyelectrolyte hydrogel by copolymerizing SBMA and AMPS with the polyethylene glycol dimethacrylate as the cross-linker, the hydrogel presented 44.5 kPa stress, 874% strain, 3.97 kPa adhesion strength. Xiong et al. [58] obtained a polymerizable rotaxane hydrogel by the photopolymerization of acrylamide with a pseudorotaxane that was formed from acrylated β-cyclodextrin and bile acid, which achieved 78.1 kPa stress, 830% strain, 4.5 kPa adhesion strength. The mechanical properties and the adhesive performance of these studies are shown in Table 1. Compared with the references, the hydrogel obtained in this study showed moderate stress (145 kPa) and strain (1580%), but it showed the highest adhesion (53.04 kPa). It is worth mentioning that the stress and strain of the hydrogel in this study are still high enough for the majority of applications under complex deformation, such as human movement. The results highlight the value of the hydrogel obtained in this study in applications.

**Figure 5:**
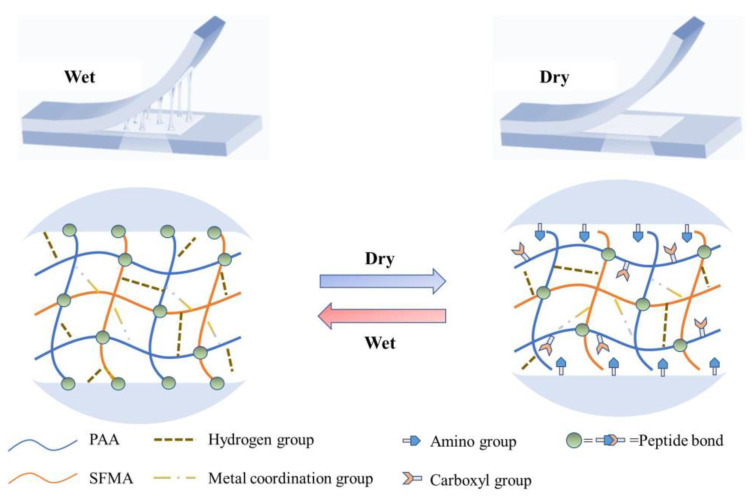
Proposed adhesion mechanism during the wet–dry transition of the SFMA/BAChol/PAA/ZnCl_2_ hydrogel.

However, the strong adhesion performance is detrimental to the carrying and transportation of the obtained hydrogel. Fortunately, the hydrogel, when freeze-dried, decreased drastically in its adhesion ability, as observed during the reversible adhesion investigation. The reversible adhesion molecular mechanism during the wet–dry transition is proposed in Figure 5. In the wet state, the hydrogel was tightly combined with the substrates with the help of hydrogen bonds, the coordination interaction, and the –NH_2_ and –COOH groups of the interface. By contrast, in the dry state, the hydrogel was rough on the surface, decreasing the interface with the substrates and increasing the interval distance. Furthermore, the Brownian movement decreased in the dry state. Both resulted in a sharp decrease in the molecule interactions between the hydrogel and the substrates. In this respect, the fresh hydrogel displayed strong adhesion, while the hydrogel after drying showed almost no adhesive ability, which is in favor of preserving and transporting the obtained hydrogel.

**Figure 6:**
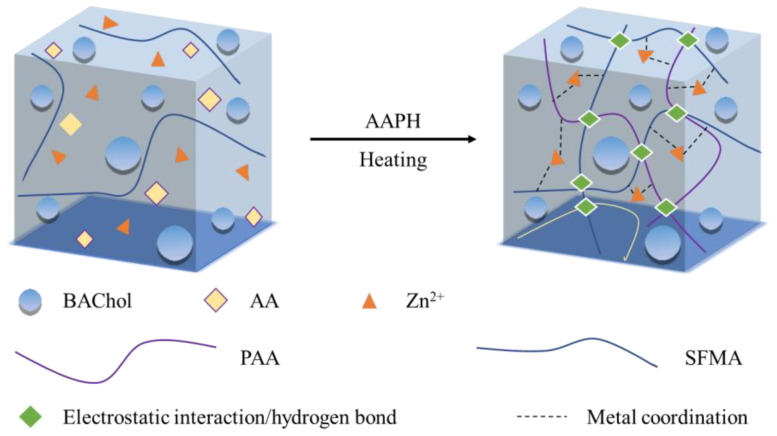
Proposed formation mechanism of the SFMA/BAChol/PAA/Zn^2+^ hydrogel.

Combining all the results and discussion in this study, the molecular formation mechanism of the hydrogel is proposed in Figure 6. When heating, the mixed solution system of SFMA, BAChol, AA, and ZnCl_2_ was copolymerized under the AAPH initiator; the main polymerizations were the self-polymerization of AA, the cross-linking between PAA chains and SFMA chains, and the self-polymerization of SFMA. The synthesized hydrogel network contained negatively charged PAA chains and SFMA chains, BAChol non-covalent interactions, Zn^2+^ ions, and Cl^-^ ions. The SFMA chains and PAA chains constituted the skeleton of the hydrogel, contributing to the good mechanical properties and the biocompatibility of the obtained hydrogel. The BAChol interacted with the other constituents through hydrogen bonds, hydrophobic associations, and coordination interactions, which constructed a second network in the hydrogel, providing the obtained hydrogel with good toughness and self-healing abilities. Three kinds of non-covalent interactions were dynamically presented in the hydrogel network, including the electrostatic interaction of PAA and SFMA chains, the hydrogen bond interaction and hydrophobic association of BAChol within and between the PAA and SFMA chains, and the coordination interaction of –COOH, –NH–, –NH_2_, and Zn^2+^ ions, which improved the involvement of PAA chains and SFMA chains and increased the structure stability of the obtained hydrogel. The dynamic balance of the non-covalent bonds and the cross-linking chains provided the obtained hydrogel with reliable self-healing capabilities and superior stretchability; the abundance of the hydroxyl and amino groups promoted the strong adhesion of the obtained hydrogel. The presence of glycerol contributed to improving the moisture retention ability and the mechanical strength because the large amount of –OH groups in the glycerol could interact through hydrogen bonds with the –NH_2_ groups, –COOH groups, and carbonyl groups from the RSFMA, PAA, and BAChol. Furthermore, the glycerol could form abundant hydrogen bonds with water molecules to retain the water in the hydrogel network and reduce moisture evaporation and freezing, contributing to the freezing-tolerant property of the hydrogel [47,48]. The presence of SFMA, BAChol, and Zn^2+^ ions contributed to the antibacterial activity, and the presence of Zn^2+^ ions also endowed the obtained hydrogel with excellent conductivity.

## 4. Methods and Materials

### 4.1. Materials and Reagents

Acrylic acid (AA), Triton X-100, and 2,2′-azobis-2-methyl-propionamidine dihydrochloride (AAPH) were bought from Macklin (Shanghai, China). ZnCl_2_, KCl, NaCl, CaCl_2_, and glycerol were purchased from Xilong Chemistry Co., Ltd., Shantou, China. *Escherichia coli* (*E. coli*) cells (CICC: 10389) were obtained from Sangon (Shanghai, China). The vinyl-modified silk fibroin (SFMA) solution was prepared in our previous study [20], which was confirmed by FT-IR spectra. The choline betulinic (BAChol) was also synthesized in our laboratory, and the chemical structure was confirmed by FT-IR and ^1^H-NMR spectra; a detailed description can be found in the Supporting Information (Section S2). All chemicals were used without further purification.

### 4.2. Preparation of the SFMA/BAChol/PAA/ZnCl_2_ Hydrogel

**Figure 7:**
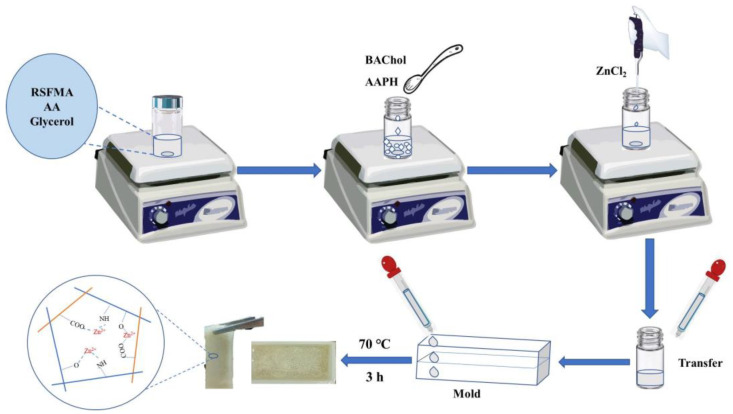
Schematic illustration of preparing SFMA/BAChol/PAA/ZnCl_2_ hydrogel.

Typically, 50 mg/mL (final concentration, the same in the following) of SFMA and 0.6 mL of AA were mixed well with 0.360 mL of glycerol, followed by adding 25 mg of BAChol under continuous stirring. Subsequently, 8 mg of AAPH (initiator) and 8 mg/mL ZnCl_2_ were added to the above solution. Following each constituent added, the solution system volume was controlled at 2.5 mL by adding extra water, and then the mixture was subjected to ultrasound for 1 min under a N_2_ atmosphere. Afterward, the mixture was transferred to a mold to react at 70 °C for 3 h under a N_2_ atmosphere, and the hydrogel was obtained. The preparation procedure is presented in Figure 7. The obtained hydrogel was characterized by various techniques, and the detailed description of the FT-IR spectra, TG curves, and SEM images can be found in the Supporting Information (Section S3).

### 4.3. Cell Culture

*E. coli* cells were cultured in the Luria–Bertani medium at 37 °C for 12 h with the help of continuous oscillation at 180 rpm. The cell suspensions were used at 1 × 10^8^ CFU/mL.

### 4.4. Informed Consent

In this study, some investigations involved human participants; these participants consented to the publication of the investigation results. The authors confirmed that the institutional review board approval was unnecessary for conducting research that simply used hydrogel samples for contact with human skin without physical modification or invasive measurement.

### 4.5. Statistical Analysis

All experiments in this study were performed in triplicate for reproducibility; the mean value was used to assess the hydrogel properties. Statistical analysis was conducted with a statistical analysis system (Origin version 9.1). The relative standard deviation in this study was ±8%.

## 5. Conclusions

Using poly-SFMA and PAA as skeletons, an SFMA/BAChol/PAA/ZnCl_2_ hydrogel was successfully fabricated with various desirable features, including superstretchability, high toughness, excellent fatigue resistance, excellent elasticity and high compressive strength, strong and reversible adhesion abilities, reliable self-healing capabilities, good electrical conductivity and conduction/strain sensitivity, good swelling and moisture retention abilities, good biocompatibility, and antibacterial activity. By varying the copolymer content, such as the amounts of SFMA, AA, BAChol, glycerol, and ZnCl_2_, the mechanical strength of the hydrogel can easily be regulated. The optimal preparation conditions were 50 mg/mL SFMA, 25 mg of BAChol, 0.6 mL of AA, 0.360 mL of glycerol, and 8 mg/mL ZnCl_2_, with 8 mg of AAPH in every 2.5 mL of the batch solution mixture. The obtained hydrogel was achieved at around 1.61 MPa at 80% compression, the maximum tensile strength was at around 0.145 MPa and the ultimate elongation at the breaking point was at around 1580%. The incorporation of BAChol greatly enhanced the toughness and self-healing performance of the obtained hydrogel owing to the formation of non-covalent bonds with the polymer skeletons. The presence of SFMA, BAChol, and Zn^2+^ ions contributed to the antibacterial activity, and the presence of Zn^2+^ ions also endowed the obtained hydrogel with excellent sensitive conductivity. The fabricated hydrogel showed multifunctional performance and demonstrated high application potential for flexible wearable sensors, achieving stable, real-time monitoring of human movement. This study provides a strategy for designing novel hydrogels with good mechanical properties and versatile functions and promotes the development of flexible sensors with stable sensitive freezing-tolerant conductivity.

## Figures and Tables

**Table 1:** Mechanical properties and adhesive performance of the hydrogels in this study and in the references.

Articles	Materials	Stress (kPa)	Strain (%)	Adhesion Strength (kPa)
Yan et al. [52]	glycerol-ionic hybrid hydrogel	5.4	15,000	6
Fan et al. [53]	poly(1-vinylimidazole)-choline chloride-glycerol eutectogel	490	2310	13.6
Yin et al. [54]	chitosan/tannic acid/poly(acrylic acid)-Al^3+^ ionic hydrogel	179.3	1450	7.02
Mo et al. [55]	tannic acid enabled dynamic interactions hydrogel	54	7300	50
Shui et al. [56]	oligo-polydopamine-tyrosine- poly(vinyl alcohol) hydrogel	13,300	813	42.1
Wang et al. [57]	zwitterionic polyelectrolyte hydrogel	44.5	874	3.97
Xiong et al. [58]	polymerizable rotaxane hydrogel	78.1	830	4.5
This study	SFMA/BAChol/PAA/ZnCl_2_ polymer hydrogel	145	1580	53.04

## Data Availability

Data are available upon request from the authors.

## Supplementary Materials

The following supporting information can be downloaded at: https://www.mdpi.com/article/10.3390/ijms252413339/s1, References [20,59] are cited in the supplementary materials.
