# Supplementary material for: Fabricating a SFMA/BAChol/PAA/ZnCl2 Hydrogel with Excellent Versatile Comprehensive Properties and Stable Sensitive Freezing-Tolerant Conductivity for Wearable Sensors
Source: Int J Mol Sci. 2024 Dec 12;25(24):13339. doi: 10.3390/ijms252413339 (PMC11728298; doi:10.3390/ijms252413339)
Supplement: Supplementary file 1 [file ijms-25-13339-s001.zip › ijms-3334837-supplementary.pdf]

Supporting Information

Fabricating a SFMA/BAChol/PAA/$ZnCl_2$ Hydrogel with Excellent Versatile Comprehensive Properties and Stable Sensitive Freezing-Tolerant Conductivity for Wearable Sensors

Jie-Ping Fan [1,2,*], Ming-Ru Xie [1], Chao Yuan [1], Jia Ma [1], Ke-Pu Fu [3], Chun-Hong Huang [3], Hui-Ping Chen [1], Hai-Long Peng [1], Chun-Fang Xie [1,2]

[1] Department of Chemical Engineering, School of Chemistry and Chemical Engineering, Nanchang University, Nanchang 330031, China

[2] Key Laboratory of Poyang Lake Ecology and Bio-Resource Utilization of Ministry of Education, Nanchang University, Nanchang 330031, China

[3] School of Basic Medical Sciences, Nanchang University, Nanchang 330006, China.

* Corresponding author.

E-mail address: jasperfan@ncu.edu.cn

S1. Effects of the preparation conditions on the hydrogel's mechanical strength

In this study, the mechanical strength under different preparation conditions was investigated; the amounts of SFMA, AA, BAchol, glycerol, and $ZnCl_2$ were optimized. In each test, the initiator AAPH was used at 8 mg, the solution system volume was controlled at 2.5 ml, and the reaction was conducted at 70 °C for 3 h under a N_2 atmosphere. The mechanical properties of the obtained hydrogel were measured at room temperature using a CMT8502 computer-controlled electronic universal tensile testing machine (Shenzhen Sansi Material Testing Co., Ltd, China). The tensile tests were performed on cuboid hydrogels (40 mm length, 20 mm width, and 1 mm thickness) and stretched at a speed of 40 mm/min. However, the effective length during tensile tests was 10 mm. The compression tests were performed on cylindrical hydrogels (20 mm diameter and 10 mm height) at a speed of 10 mm/min.

With 7.5 mg of BAChol, 0.5 ml of AA, 0.415 ml of glycerol, and 8 mg/ml $ZnCl_2$, the effect of the SFMA amount was investigated, varying from 30 mg/ml to 70 mg/ml, and the results are shown in Figure S1. By increasing the SFMA amount, both the strain and the stress of the hydrogel first increased and then decreased. At a low SFMA amount, the cross-linking degree was increased by increasing the SFMA, contributing to a tighter hydrogel network; therefore, the mechanical strength of the obtained hydrogel increased. However, when too much SFMA was used, the cross-linking degree was too high on the hydrogel network structure, resulting in the stiffness of the obtained hydrogel, and the elongation at the breaking point drastically decreased. The optimal SFMA amount was 50 mg/ml.

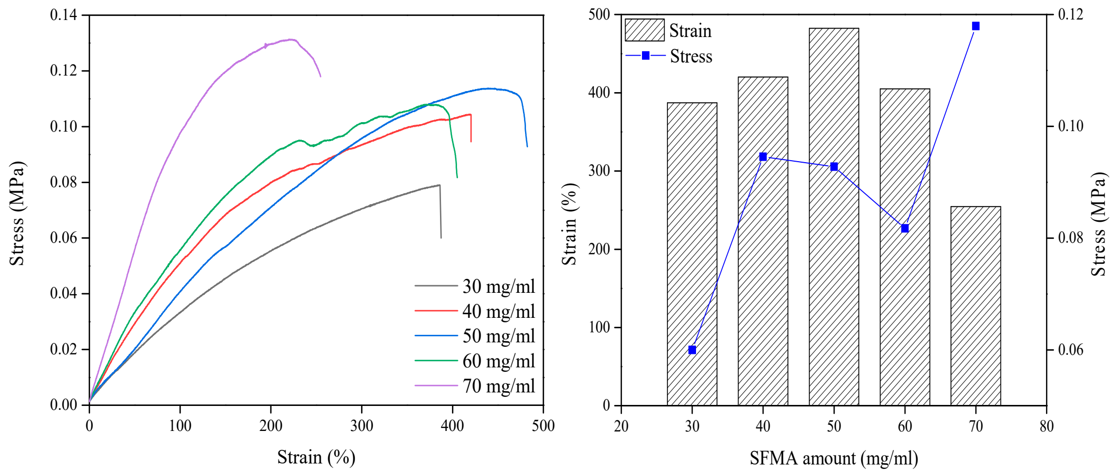

Figure S1. Effect of SFMA amount on hydrogel mechanical strength.

The effect of AA on the hydrogel mechanical strength was investigated, varying from 0.4 ml to 0.8 ml, at 50 mg/ml SFMA, 7.5 mg of BAChol, 0.415 ml of glycerol, and 8 mg/ml $ZnCl_2$. The results are shown in Figure S2; the stress and strain of the hydrogel increased with the increase in the AA amount from 0.4 ml to 0.6 ml and then decreased by further increasing the AA amount. When too little AA was used, the cross-linking degree of the hydrogel network was low, and the obtained hydrogel was fragile. With the AA amount increased, the hydrogel network became stronger, and the soft hydrogel was obtained. Further increasing the AA amount, the mechanical strength of the obtained hydrogel was reduced; the network of the obtained hydrogel was tight under a high AA content, and the softness of the obtained hydrogel decreased. The optimal AA amount was 0.6 ml.

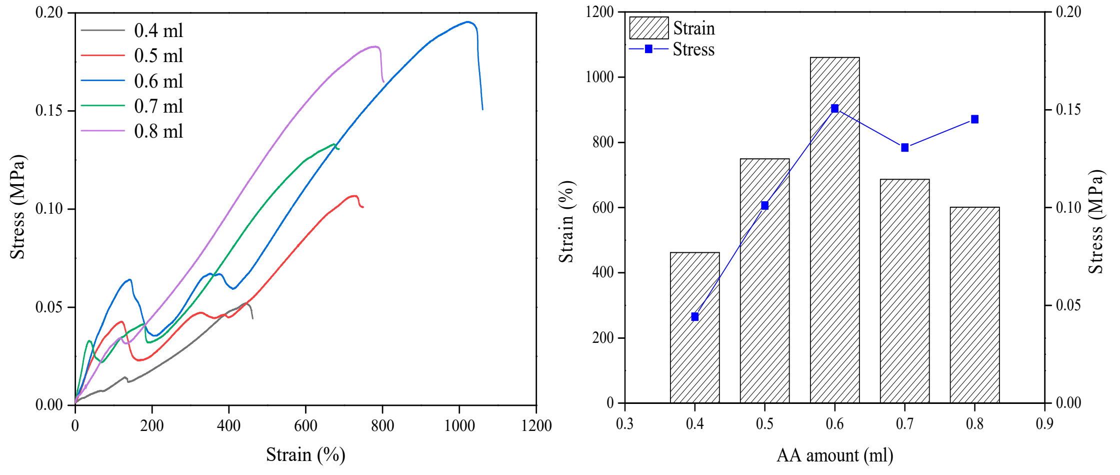

Figure S2. Effect of AA amount on hydrogel mechanical strength.

The effect of BAChol on the hydrogel mechanical strength was investigated, varying from 0 mg to 50 mg, with a final concentration of 0 mg/ml-20 mg/ml, at 50 mg/ml SFMA, 0.6 ml of AA, 0.415 ml of glycerol, and 8 mg/ml $ZnCl_2$. The results are shown in Figure S3; by increasing the BAChol amount, the stress of the obtained hydrogel increased within the whole investigated range, while the strain increased from 0-25 mg (0-10 mg/ml) and then decreased from 25-50 mg (10-20 mg/ml). The non-covalent bonding interaction was stronger at a high BAChol amount, achieving a higher stress of the obtained hydrogel. By contrast, at a low BAChol amount, the physical cross-linked network was unstable. However, at a high BAChol amount, the toughness of the obtained hydrogel decreased because the strength of the hydrogel was too high. The optimal BAChol amount was 25 mg, i.e., 10 mg/ml.

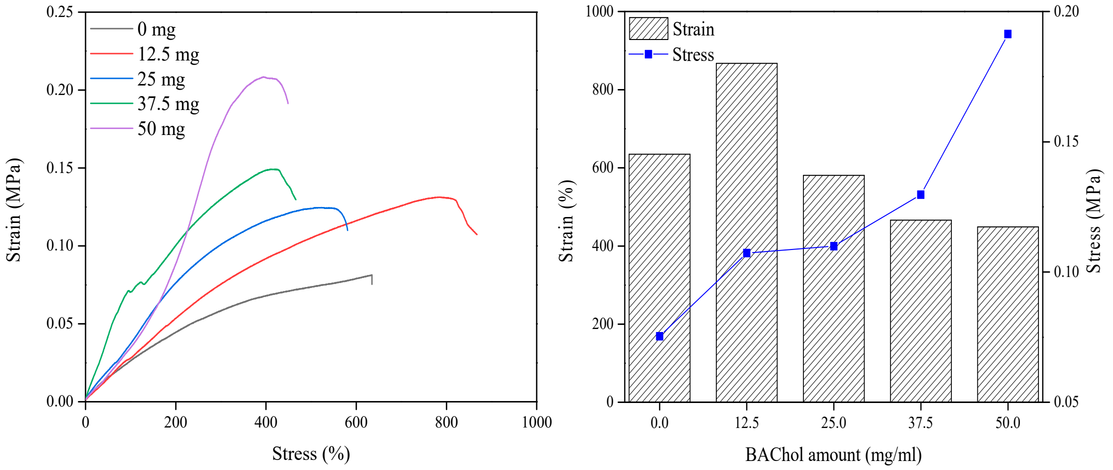

Figure S3. Effect of BAChol amount on hydrogel mechanical strength.

The effect of glycerol on the hydrogel mechanical strength was investigated, varying from 0.310 ml to 0.625 ml, with a proportion of 1:7-1:3 in the solution mixture, at 50 mg/ml SFMA, 25 mg of BAChol, 0.6 ml of AA, and 8 mg/ml $ZnCl_2$. The results are shown in Figure S4; both the strain and stress of the hydrogel increased and then decreased, with the highest value achieved for the 1:6 proportion. The addition of glycerol was able to improve the hydrogel moisturizing properties, and the –OH groups in glycerol could interact through hydrogen bonds with the $–NH_2$ and –COOH groups in SFMA, PAA, and BAChol; both contributed to improving the hydrogel network's tightness and the hydrogel's mechanical strength. However, when

too much glycerol was used, the metal coordination interaction was disturbed by excessive glycerol, the polymer chains became fragile, and the mechanical strength of the obtained hydrogel decreased. The optimal glycerol amount was 0.415 ml with a proportion of 1:6 in the solution mixture.

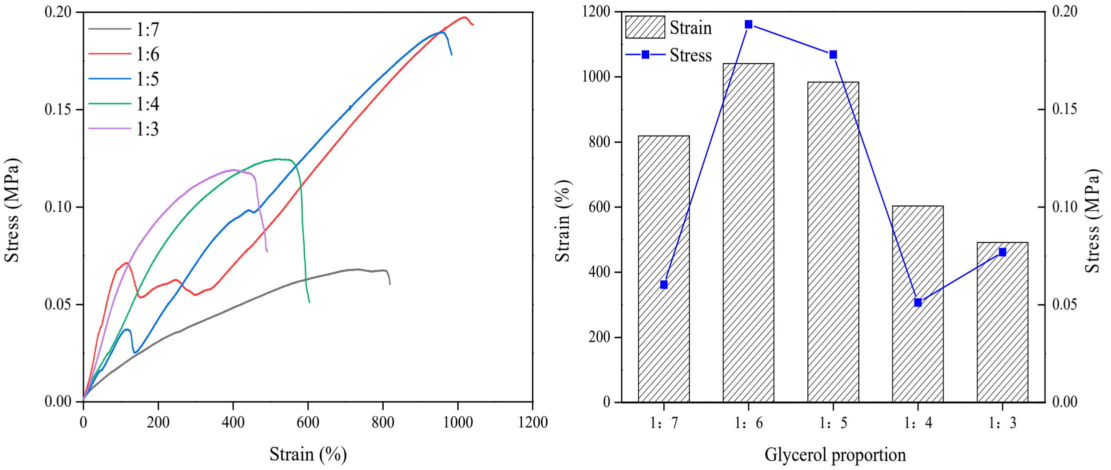

Figure S4. Effect of glycerol amount on hydrogel mechanical strength.

The effect of $ZnCl_2$ on the hydrogel mechanical strength was investigated, varying from 4 mg/ml to 20 mg/ml (final concentration), at 50 mg/ml SFMA, 25 mg of BAChol, 0.6 ml of AA, and 0.360 ml of glycerol. The results are shown in Figure S5; by increasing the $ZnCl_2$ amount, the Zn^{2+} coordination interaction improved and the hydrogel network became tighter, so the mechanical strength of the obtained hydrogel increased. However, at a high Zn^{2+} concentration, the AA polymerization was prevented by Zn^{2+}, resulting in a decrease in the cross-linking degree and a loose structure of the obtained hydrogel network, so the mechanical strength of the obtained hydrogel decreased. Considering the value of strain and stress, the optimal $ZnCl_2$ amount was 8 mg/ml (final concentration) in this study.

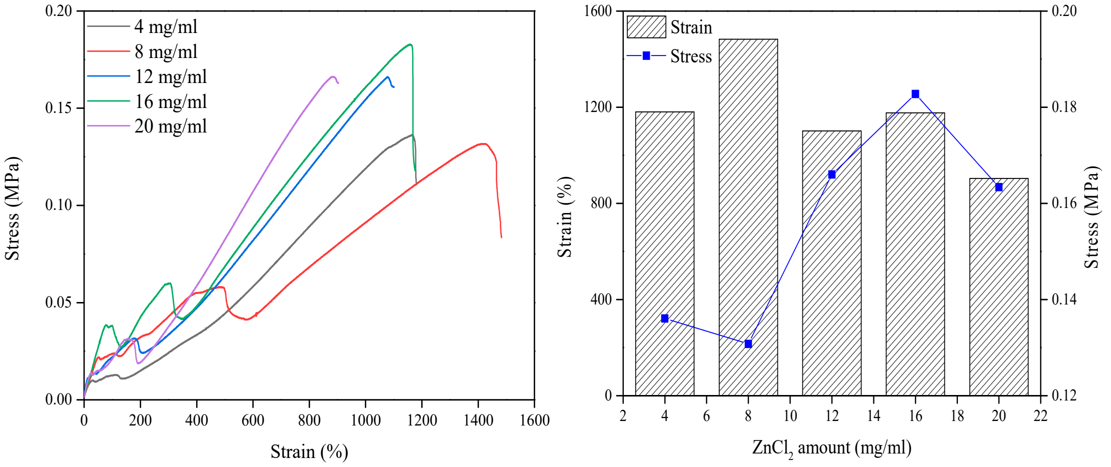

Figure S5. Effect of $ZnCl_2$ amount on hydrogel mechanical strength.

According to the above discussion, the optimal preparation conditions were 50 mg/ml SFMA, 25 mg of BAChol, 0.6 ml of AA, 0.360 ml of glycerol, and 8 mg/ml $ZnCl_2$, with 8 mg of AAPH in every 2.5 ml of the batch solution mixture. Under these conditions, the hydrogel was prepared, and the obtained hydrogel was subjected to mechanical strength testing; the straightforward observations of the compression and tensile tests are shown in Figure S6. The mechanical strength of the obtained hydrogel was achieved at around 1.61 MPa at 80% compression. During the tensile test, the maximum tensile strength of the obtained hydrogel was achieved at around 0.145 MPa; the elongation at the breaking point was around 1580%.

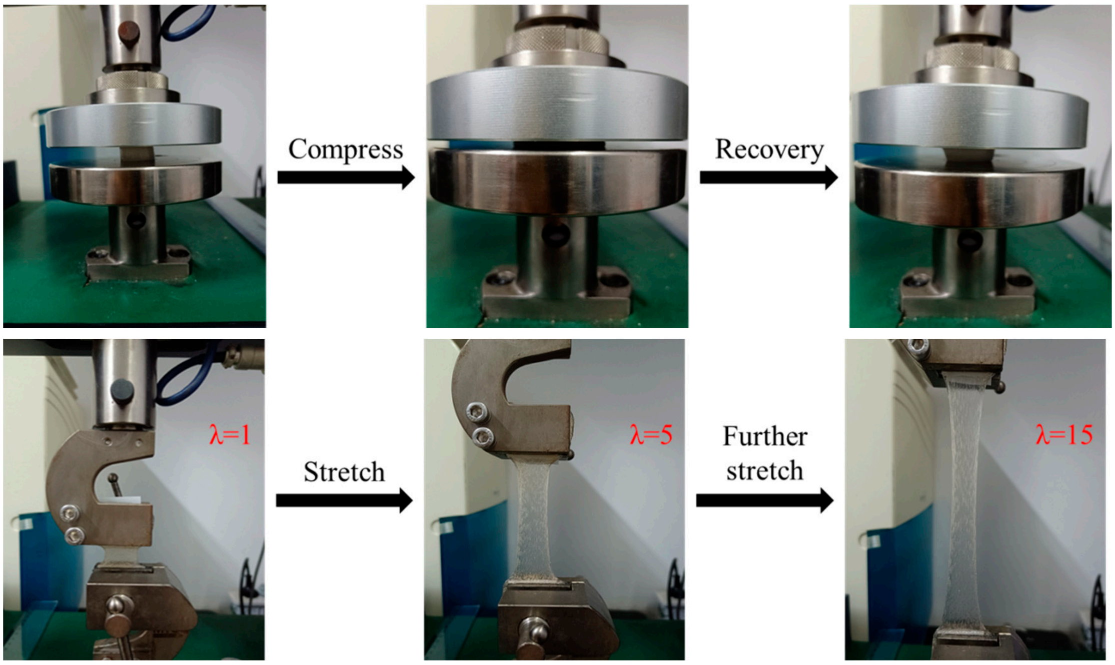

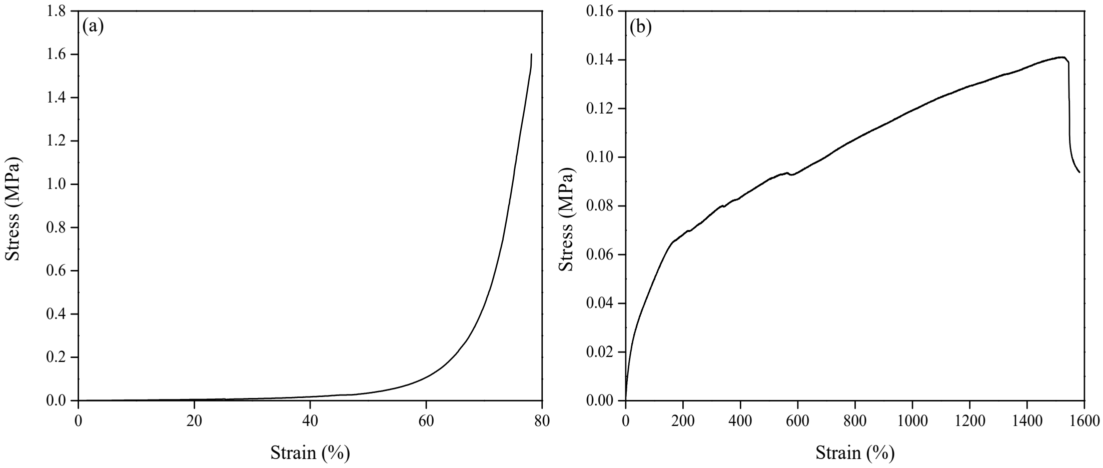

Figure S6. Optical images demonstrating the strength of the obtained hydrogel under optimal preparation conditions and the stress–strain curves for the hydrogel under uniaxial compression (a) or tension (b).

S2. Synthesis of choline betulinic

To synthesize choline betulinic (BAChol), choline chloride was subjected to anion-exchange chromatography using methanol as the solvent, and the alkaline eluent ([Chol][OH]) was collected. The possible chlorine in the eluents was detected by $AgNO_3$ (HNO_3), and no precipitation was observed, indicating the absence of chlorine. The obtained [Chol][OH]/methanol solution was titrated by standard HCl to determine the [Chol][OH] concentration. Afterward, an equimolar amount of betulinic acid (BA) was added to the [Chol][OH] solution and reacted at 50 °C for 4 hours. BAChol was obtained by the evaporation of the extra solvent. The obtained BAChol was dried at 60 °C for 48 h and crushed for further use. The synthesis procedures are presented in Figure S7. The FT-IR spectra of BA and BAChol are shown in Figure S8. For the BA spectra, the peak at around 1690 cm^{-1} was assigned to the stretching vibration of C=O from the –COOH group. For the BAChol spectra, the peaks at around 1550 cm-1 and 1370 cm-1 were assigned to the asymmetric stretching vibration and symmetric stretching vibration of C=O from the ester bond, respectively. The disappearance of the C=O peak of the –COOH group and the clearly detected C=O peaks of the ester bond indicated the successful synthesis of BAChol from BA and choline chloride. Furthermore, the ^{1}H-NMR analysis of BA and BAChol was performed on an NMRready 60 pro spectrometer at 400 MHz using DMSO-d6 as the solvent. The results are shown in Figure S9; the peak at δH 12.02 ppm was assigned to the hydrogen atom from the –COOH group in BA, the peaks at δH 4.53 ppm and δH 4.61 ppm were assigned to the hydrogen atoms from the olefin bond, and the peak at δH 3.78 ppm was assigned to the hydrogen atom from the –CH_2– group in BAChol. Comparing the two spectra, the peak at δH 12.02 ppm disappeared in the BAChol spectra, and the peak area of the hydrogen atoms from the olefin bond was almost the same as the peak area of the peak at δH 3.78 ppm, indicating the successful synthesis of BAChol from BA and choline chloride. Both FT-IR spectra and ^{1}H-NMR spectra confirmed that BAChol was synthesized successfully.

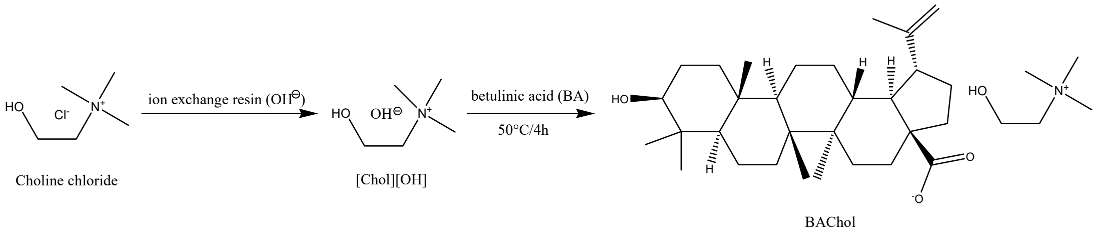

Figure S7. Schematic strategy of BAChol synthesis.

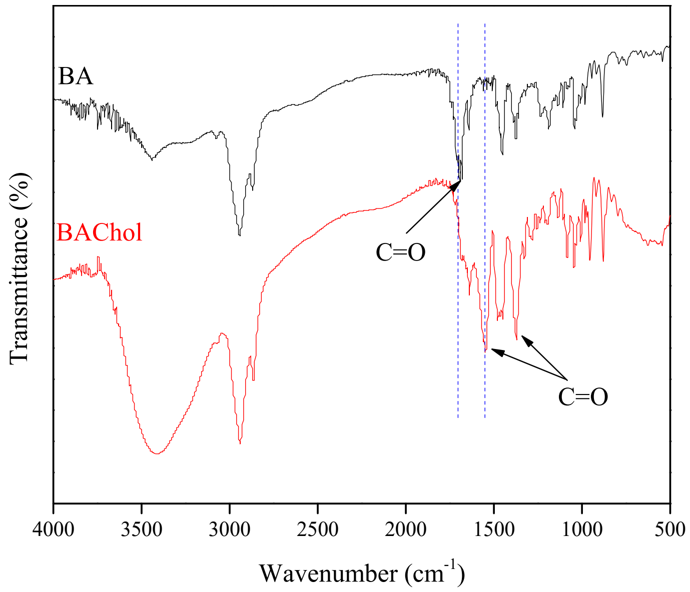

Figure S8. FT-IR spectra of BA and BAChol.

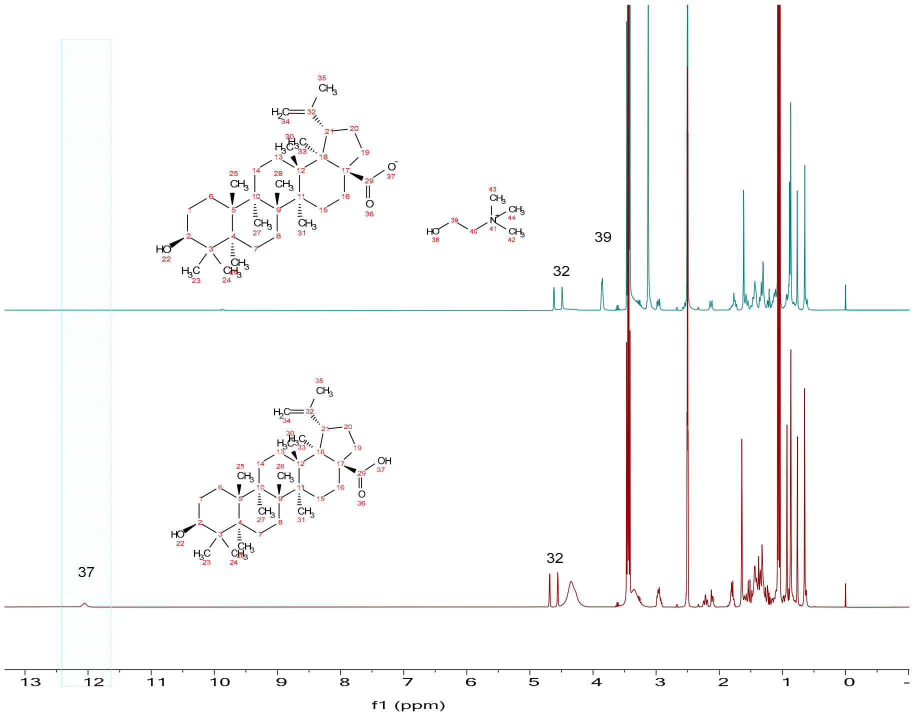

Figure S9. ^{1}H-NMR spectra (400 MHz, DMSO-d6) of BA and BAChol.

S3. Hydrogel characterization

The ATR-FT-IR spectra of the lyophilized hydrogel were acquired on a Thermo Scientific Nicolet 5700 FT-IR spectrometer at a resolution of 4 cm^{-1} with micro-ATR accessories. The FT-IR spectra (4000–400 cm^{-1}) results are shown in Figure S10; the broad peak at around 3300 cm^{-1} was attributed to the –OH stretching vibration. For the SFMA spectra, the peak at around 1654 cm^{-1} was assigned to the C=O stretching vibration (amide I), the peak at around 1546 cm^{-1} was assigned to the C–N stretching vibration coupled with the N–H bending vibration (amide II), the peak at around 1234 cm^{-1} was assigned to the C–N stretching vibration coupled with the N–H bending vibration (amide III), and the peak at around 945 cm^{-1} was assigned to the –C=CH_2 stretching vibration of the methacrylate vinyl group [1]. For the BAChol spectra, the intense broad peak at around 2936 cm^{-1} was assigned to the C–H stretching vibration of the –CH_3 group, and the peaks at around 1546 cm^{-1} and 1372 cm^{-1} were assigned to the C=O stretching vibration in carboxylate. In the hydrogel spectra, the peak at around 1710 cm^{-1} was assigned to the –COOH absorption in PAA [2]. Furthermore, the characteristic peaks of SFMA and BAChol can be clearly observed in the hydrogel spectra, and the peak at around 2949 cm^{-1} was assigned to the formation of hydrogen bonds. The results demonstrated the successful synthesis of the hydrogel by SFMA, PAA, and BAChol. Further comparing the spectra of the SFMA/BAChol/PAA/$ZnCl_2$ hydrogel with the SFMA/BAChol/PAA hydrogel, the peak that appeared at around 1616 cm^{-1}, together with the obvious peaks shift, was attributed to the coordination interaction under the Zn^{2+}.

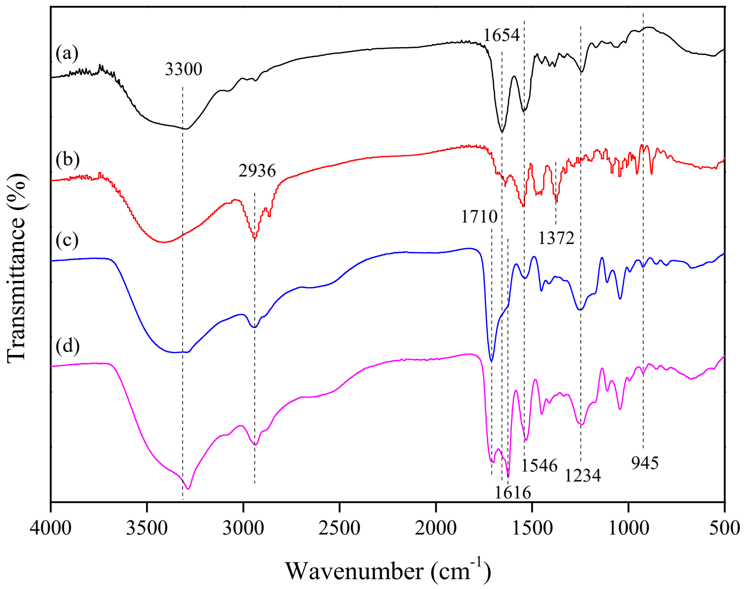

Figure S10. FT-IR spectra of SFMA (a), BAChol (b), SFMA/BAChol/PAA hydrogel (c), and SFMA/BAChol/PAA/$ZnCl_2$ hydrogel (d).

TG data were collected by using a thermal gravimetric analyzer (SDT Q600, TA Instruments, USA) at a heating rate of 10 °C/min under a 100 ml/min N_2 atmosphere. The TG curves of SFMA, BAChol, SFMA/BAChol/PAA hydrogel, and SFMA/BAChol/PAA/$ZnCl_2$ hydrogel are shown in Figure S11. For all four, the initial weight loss at 25-150 °C was attributed to the adsorbed and bound water weight loss. For SFMA, the weight loss at 220-360 °C was assigned to the degradation of polymer chains, while that at 360-600 °C was ascribed to carbonization. For BAChol, the weight loss at 200-230 °C was assigned to choline skeleton degradation, and the weight loss at 250-400 °C was assigned to betulinic skeleton degradation. Compared to the weight loss of SFMA and BAChol, the weight loss of SFMA/BAChol/PAA hydrogel displayed the same tendency as that of SFMA and BAChol, indicating the structure skeleton of SFMA and BAChol in the obtained SFMA/BAChol/PAA hydrogel. Furthermore, the multiple weight loss at 300-500 °C in the SFMA/BAChol/PAA hydrogel might be attributed to the physical crosslinking network degradation, the chemical crosslinking network degradation, and the glycerol degradation, respectively. However, when regarding the SFMA/BAChol/PAA/$ZnCl_2$ hydrogel, the weight loss at 300-500 °C became a smooth and continuous curve,

which was assigned to the network degradation of the interconnected physical crosslinking and chemical crosslinking. The results indicated that the independent physical crosslinking network and chemical crosslinking network in the SFMA/BAChol/PAA hydrogel were dynamically integrated by the Zn^{2+} coordination interaction, contributing to the stability and toughness of the hydrogel structure.

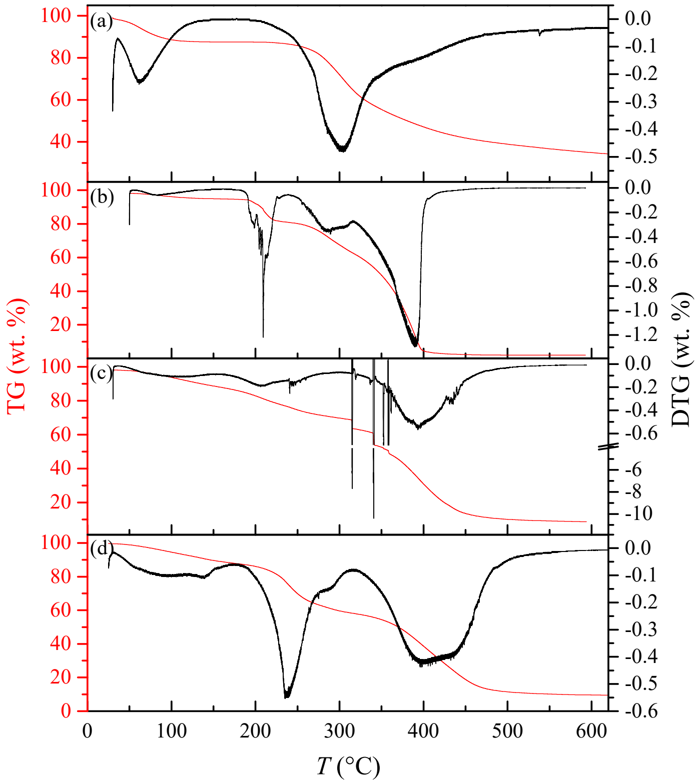

Figure S11. TG and DTG curves of SFMA (a), BAChol (b), SFMA/BAChol/PAA hydrogel (c), and SFMA/BAChol/PAA/$ZnCl_2$ hydrogel (d).

The morphology and structure of the lyophilized hydrogel were acquired on a field-emission scanning electron microscope (SEM, JEOL, JSM-6701F). The SEM images of the SFMA/BAChol/PAA hydrogel and the SFMA/BAChol/PAA/$ZnCl_2$ hydrogel, together with the energy dispersive X-ray spectroscopy (EDS) point analysis of the SFMA/BAChol/PAA/$ZnCl_2$ hydrogel, are shown in Figure S12. Both hydrogels displayed a three-dimensional network structure. Compared with the SFMA/BAChol/PAA hydrogel, the SFMA/BAChol/PAA/$ZnCl_2$ hydrogel was denser and more compact in structure, which resulted from the coordination interaction of Zn^{2+} and was in favor of improving the mechanical properties of the hydrogel. The

EDS results also proved the presence of Zn^{2+} in the SFMA/BAChol/PAA/$ZnCl_2$ hydrogel.

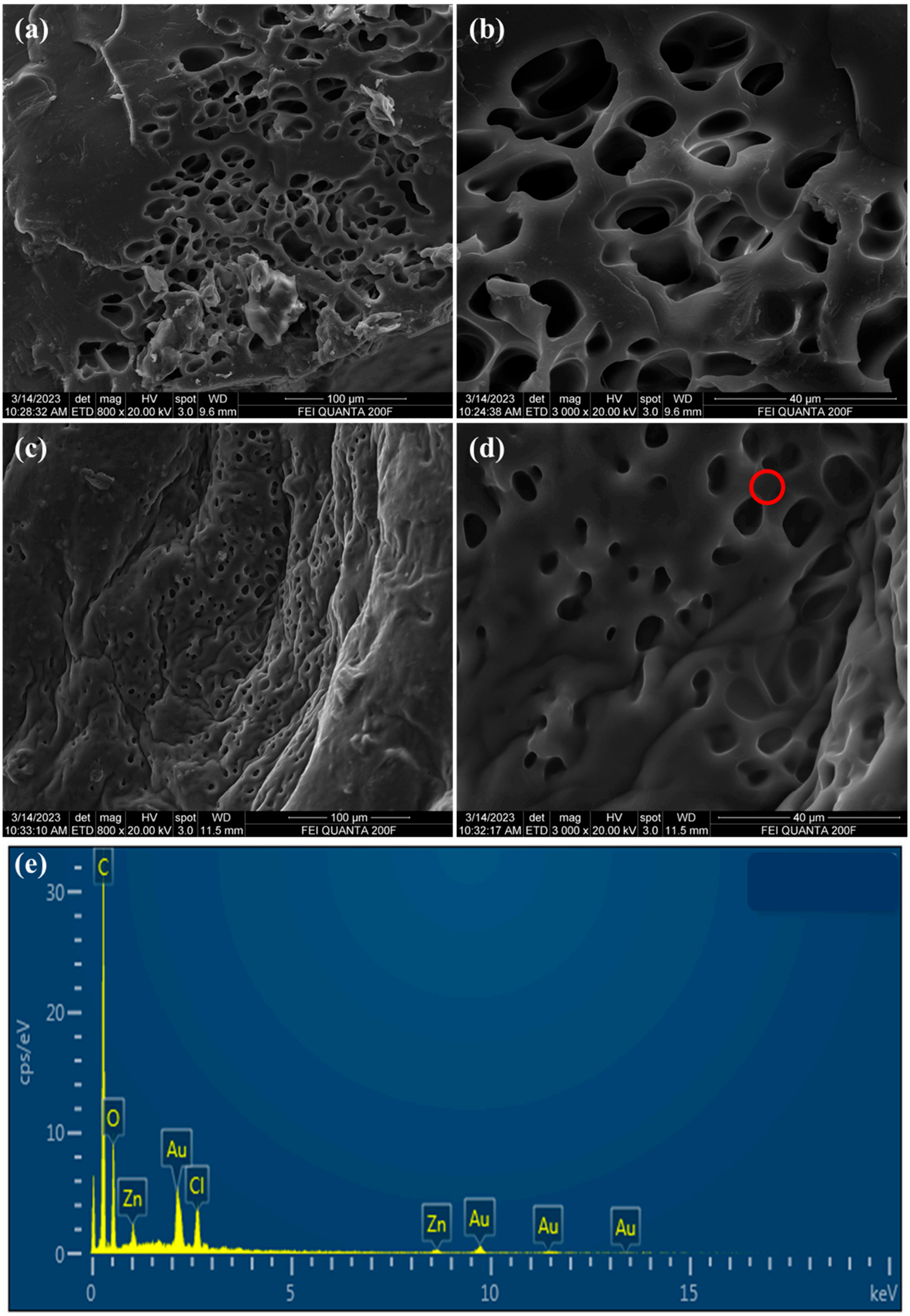

Figure S12. SEM images of the SFMA/BAChol/PAA hydrogel ((a) and (b)) and the

SFMA/BAChol/PAA/$ZnCl_2$ hydrogel ((c) and (d)); (e) is the EDS result of the selected area in (d).

References

1. Fan, J.-P.; Tian, J.-M.; Zhong, H.; Chen, H.-Q.; Xie, C.-F.; Chen, H.-P.; Peng, H.-L.; Liu, Y.-D. Synthesis of a porous hollow magnetic molecularly imprinted microsphere by O/W/O composite emulsion polymerization for specifically recognizing bovine serum albumin. *Separation and Purification Technology* **2024**, *329*, 125197, doi:https://doi.org/10.1016/j.seppur.2023.125197.
2. Hu, Y.; Jiang, X.; Ding, Y.; Ge, H.; Yuan, Y.; Yang, C. Synthesis and characterization of chitosan–poly(acrylic acid) nanoparticles. *Biomaterials* **2002**, *23*, 3193-3201, doi:https://doi.org/10.1016/S0142-9612(02)00071-6.
